# Sex-Dependent Modulation of AMPA and GABA_A_ Receptors in Response to Perinatal Stress: Implications for Cognitive and Emotion-related Behaviors

**DOI:** 10.2174/011570159X328731250104062122

**Published:** 2025-06-02

**Authors:** Alessandra Gaetano, Vance Gao, Gilles Van Camp, Hammou Bouwalerh, Milena Cannella, Tiziana Imbriglio, Sergio Scaccianoce, Stefania Maccari, Sara Morley-Fletcher

**Affiliations:** 1 Univ. Lille, CNRS, UMR 8576, UGSF, Unité de Glycobiologie Structurale et Fonctionnelle, GlycoStress Team, F-59000 Lille, France;; 2 Univ. Lille, Inserm, CHU Lille, Institut Pasteur de Lille, U1011- EGID, F-59000 Lille, France;; 3 IRCCS Neuromed, Pozzilli (IS), Italy;; 4 Department of Physiology and Pharmacology “V. Erspamer”, University Sapienza of Rome, Rome, Italy;; 5 Department of Science and Medical-Surgical Biotechnology, University Sapienza of Rome, Rome, Italy;; 6 LIA-CNRS, International Associated Laboratory (LIA), France, Italy “Perinatal Stress and Neurodegenerative Diseases” ULille - CNRS, UMR 8576, and Sapienza UniRome1 - IRCCS Neuromed, Lille, France

**Keywords:** Perinatal Stress (PRS) animal model, prefrontal cortex, dorsal and ventral hippocampus, glutamate/GABA, cognition and risk-taking behaviors, behavioral response to novelty, adverse environment

## Abstract

**Background:**

Early-life stress can severely impact brain health and neuronal plasticity, potentially leading to psychiatric disorders, with excitatory and inhibitory neurotransmission changes being key to understanding and mitigating these effects.

**Objective:**

We investigated the effects of Perinatal Stress (PRS) on the balance of excitatory and inhibitory neurotransmission, particularly focusing on AMPA and GABA_A_ receptor protein levels and their relationship with cognition and risk-taking behavior in male and female Sprague-Dawley rats.

**Methods:**

Adult PRS (3-4 months old) offspring of dams exposed to 10 days of gestational restraint stress, which led to reduced maternal care, were evaluated at 3-4 months for behavioral responses to novelty, adverse environments, and recognition memory, with biochemical analyses conducted in the prefrontal cortex and the ventral and dorsal hippocampus.

**Results:**

PRS and sex notably affected behavior and AMPA/GABA_A_ receptor subunit expression. PRS males showed reduced risk-taking behavior when exposed to novel and adverse environments and impaired recognition memory, while PRS females demonstrated better behavioral performance compared to both PRS males and control females. In the dorsal hippocampus, PRS increased the GluA2:GluA1 ratio and GABAA-α1 subunit in females but reduced them in males, modulating the AMPA/GABAA balance to enhance synaptic GABAergic inhibition and behavioral resilience in PRS females and control males.

**Conclusion:**

Our findings indicate that increased synaptic inhibition and reduced excitatory noise may underlie enhanced recognition memory and risk-taking behavior. The sex differences in PRS rats suggest that targeting AMPA or GABA_A_ receptors could help treat early-life stress-related disorders and underscore the need for developing gender-specific therapies.

## 1 INTRODUCTION

Maladaptive family functioning, neglect, and low maternal care, during the perinatal period make individuals more vulnerable to psychiatric problems later in life and may reduce the lifespan of adult offspring [1, 2]. Several epidemiological studies have reported that children born to mothers who experienced stress during pregnancy and the postpartum period show delays in cognitive development, problems in social relationships, alterations in emotional behavior, and higher rates of suicide later in life [3, 4]. These negative outcomes, documented both clinically and preclinically, are associated with changes in the volume and morphology of stress-sensitive brain regions (*i.e*., hippocampus, prefrontal cortex, and amygdala), regulating memory, executive functions, and emotion-related behavior such as response to novelty and adverse environments [5]. Furthermore, these brain regions still developing during the critical perinatal period could be strongly influenced by early-life stress (ELS). Indeed, ELS events have profound effects on brain health, causing persistent changes in behavior, synaptic organization, and neuronal plasticity later in life caused by the intervention in the brain developing period [5-8].

To date, research on the impact of maternal stress on offspring development is continuously evolving and requires the use of various animal models for a clear understanding of its molecular picture and for the development of consequently targeted interventions and prevention strategies [9]. Reduced risk-taking behavior in response to novelty and adverse environments, increased immobility time in the forced swim test, anhedonia in the sucrose preference and splash tests, impaired learning and memory, and constitute the spectrum of behavioral changes observed in offspring exposed to ELS [5, 10]. These behavioral patterns have been consistently observed in well-known and robust animal models of ELS (*e.g*., perinatal stress, maternal separation, limited bedding) which are used to explore the neuroendocrine and behavioral impact of early adverse events [6, 11, 12]. Despite the implementation of different protocols, a common denominator of all models of ELS is a lasting dysregulation of stress hormone secretion, caused by changes in the activity of the hypothalamic-pituitary-adrenal (HPA) axis and the stress response [10]. These behavioral and neuroendocrine changes are associated with disruptions in the adrenergic, serotonergic, and dopaminergic neurotransmission systems, which have been proposed as potential molecular mechanisms of the observed patterns in both animal models and humans [13-17]. Specifically, human and rodent offspring with a history of ELS (*e.g*., reduced parental care, maternal separation) display increased ventral striatum dopamine levels in response to a psychological stress task and, increased consumption of psychostimulants and drugs during adulthood [17]. Furthermore, animal studies have demonstrated that exposure to both acute and chronic stress during critical developmental periods leads to significantly altered connectivity in glutamatergic and GABAergic transmissions within hippocampal and cortical circuits [8, 18-21]. Indeed, the down-regulation of the kainate glutamate ionotropic receptors-modulation, which regulates GABAergic transmission, contributes to the decreased reactivity to novelty in the open field after maternal separation [22]. Moreover, it has been reported that ELS can both accelerate [23] or delay [24] the GABA-switch and the maturation of excitatory/inhibitory balance, leading to a deficit in neuronal activity. These findings on glutamate/GABA balance highlight the need to better understand how changes in glutamatergic and GABAergic neurotransmission contribute to balance disruptions in the pathophysiology of stress-related disorders in early life. Within this context, the Perinatal Stress (PRS) model in rats, where pregnant rats are subjected to repeated restraint stress resulting in impaired maternal behavior [10], displays a pattern where the modulation of glutamatergic neurotransmission is at the core of consistent behavioral pathological phenotype observed in the offspring. Particularly, our investigations have unveiled a reduction in depolarization-evoked glutamate release specifically within the ventral hippocampus [25-27], a hippocampal subregion intricately linked to behavioral and biological stress response [28]. Notably, our research has further revealed that local administration of a pharmacological cocktail comprising blockers of mGlu2/3 metabotropic glutamate receptors and GABA_B_ receptors effectively restored glutamate release and increased risk-taking behavior in response to novelty in PRS male rats [25]. Furthermore, we have found that chronic treatment with a selective positive allosteric modulator of AMPA receptors, normalized hippocampal mGlu2/3 receptors and corrected abnormalities in risk-taking, motivational, and cognitive behaviors in adult PRS rats [29]. PRS modulates glutamate receptors in different brain regions correlated to cognition and emotion-related behaviors up to ageing, showing long-term programming of ELS on glutamatergic receptors [30]. This evidence strongly suggests that an aberrant balance between excitatory and inhibitory neurotransmission in cortical and hippocampal circuits underlies PRS programming effects on the developmental trajectory of offspring exhibiting a pathological phenotype throughout the life course.

Changes in the expression and composition of AMPA and GABA_A_ receptor subunits are largely unknown in the PRS model, and given the distinct roles of AMPA and GABA_A_ receptor subunits, it is reasonable to anticipate their involvement in the programming induced by PRS. AMPA and GABA_A_ ionotropic receptors play pivotal roles in mediating rapid excitatory and inhibitory synaptic transmission within the CNS. AMPA receptors are ion channels activated by glutamate and are constructed through the assembly of four subunits known as GluA_1_ to GluA_4_. The presence of the GluA_2_ subunit within this heterotetrameric structure serves as a gatekeeper, preventing the passage of calcium ions (Ca^2+^) through the channel. Consequently, over 90% of AMPA receptors selectively allow the influx of sodium ions (Na^+^). It is important to note that the quantity, subunit composition, and activity of AMPA receptors localized on dendritic spines critically influence the efficiency of excitatory synaptic transmission. Moreover, changes in AMPA receptor functionality constitute the underlying mechanisms of activity-dependent synaptic plasticity, encompassing phenomena like long-term potentiation (LTP) and long-term depression (LTD) [31-33]. GABA_A_ receptors, functioning as ligand-gated anion channels, assemble heteropentamerically, with subunits including α_1-6_, β_1-3_, γ_1-3_, δ, ε, θ, or π. In mature neurons, GABA_A_ receptors permit chloride ion (Cl^-^) influx, following the concentration gradient generated by the K^+^/Cl^-^ symporter, KCC2, which effectively extrudes Cl^-^ from the cell, thus maintaining low intracellular Cl^-^ concentrations. Counteracting KCC2, the Na^+^/K^+^/Cl^-^ cotransporter, NKCC1, predominates in immature neurons and facilitates Cl^-^ transport into the cell [34-40]. Within the synaptic milieu, GABA_A_ receptors predominantly consist of α_1_, α_2_, α_3_, and γ_2_ subunits, making them targets for benzodiazepines. In contrast, extra-synaptic GABA_A_ receptors incorporate α_4_, α_5_, α_6_, and δ subunits, rendering them susceptible to modulation by ethanol, neurosteroids, and general anesthetics. Notably, extra-synaptic GABA_A_ receptors exhibit a high affinity for GABA and mediate tonic synaptic inhibition within the CNS [41].

Thus, in this study, we delved into the impact of PRS on the expression of GluA_1_, GluA_2_, and GluA_3_ AMPA receptor subunits, as well as α_1_, α_2_, α_4_, γ_2_, and δ GABA_A_ receptor subunits, alongside KCC2 and NKCC1, within the prefrontal cortex, the ventral and dorsal hippocampus of both male and female adult rats. Additionally, we examined cognitive and emotion-related behavior in response to novelty mediated by these brain regions using the 2-chamber apparatus and the Elevated Plus Maze (EPM), in order to determine the potential associated mechanism of the developmental programming induced by PRS.

## 2 MATERIALS AND METHODS

### 2.1 Ethics

All experiments were carried out in accordance with the NC3Rs ARRIVE Guidelines [42] and followed the rules of European Communities Council Directive 2010/63/EU. The local Committee CEEA-75 (*Comité d’Ethique en Experimentation Animale Nord-Pas de Calais, 75*) approved the experimental procedures (research question, key design features, and analysis plan) that were carried out under the MESRI (Ministère de l’Enseignement supérieur, de la Recherche et de l'Innovation) registration number (#33654). No human endpoints were set in the present study.

### 2.2 Animals

28 nulliparous female Sprague-Dawley rats, weighing approximately 250 g, were purchased from Charles River (France) and group-housed (five females/cage) in individually ventilated cages (IVCs; Tecniplast, Italy) and kept under standard conditions at a temperature 21-22°C, humidity of 50% to 60%, with a 12 h light/dark cycle (lights on 7 am: lights off 7 pm) with ad libitum access to food and water. After group housing for two weeks, each female was individually housed for one week with a sexually experienced male rat. Following that, a gain of at least 10 grams was considered as an index of pregnant status. As bedding, a mixture of sawdust and cellulose was provided and a chewable food stick was added as minimal environmental enrichment condition. During gestation, enrichment was improved by adding 20 gr of cellulose during the last week of gestation as nesting material.

### 2.3 Perinatal Stress (PRS) Procedure

Half of the pregnant females (n = 14) were randomly assigned to a stress procedure, which was conducted according to our standard protocol [43], as illustrated in Fig. (**1**). Briefly, from day 11 of gestation until delivery, pregnant females were subjected to restraint in a transparent plastic cylinder and exposed to bright light during three daily sessions of 45 minutes. During the stress procedure, females were inspected daily for their well-being and any expected or unexpected adverse events (*i.e*. abrupt drop of body weight sign of spontaneous abortion or degradation of fur) were recorded by the animal caregivers and the experimenters. Control pregnant females were left undisturbed in their home cages and were handled only once per week. Since gestational stress induces a reduction in maternal behavior [44] (less than 40% of maternal behavior observed in stressed mothers), we refer to the entire procedure as Perinatal Stress (PRS). All pregnant females subjected to the stress procedure [42] exhibited reduced maternal behavior. After weaning, male and female offspring from litters with a balanced sex ratio were used for the experiments. Animals were housed in pairs or groups of three under consistent environmental conditions throughout the experiments, conducted when they were 3-4 months old.

### 2.4 Experimental Design

The groups compared were as follows: CONT and PRS, each consisting of rats of both sexes (males and females). Within each experimental group (CONT and PRS, males and females), a maximum of two rats from the same litter were selected to avoid the litter effect. Importantly, animals selected for biochemical analysis did not undergo any behavioral testing. Behavioral testing and protein level measurements were performed in distinct groups of rats to ensure that biochemical data were not influenced by the learning paradigm. Sample size estimates were based on prior behavioral and biochemical studies conducted in our lab, calculated using STATISTICA software, with the power of the experiment set at 80%. While a minimum of 6 rats per group was initially planned, this target was not fully achieved due to challenges with behavioral recording and tissue collection for biochemical analysis. Ultimately, a total of 70 animals were used, divided as follows: Set 1: Spatial Recognition Test - 22 rats, Set 2: Elevated Plus Maze (EPM) Test - 25 rats, Set 3: Biochemical Analysis - 23 rats. For behavioral data, each animal served as the experimental unit, whereas for biochemical data, the hippocampus (ventral and dorsal, right and left) and prefrontal cortex (right and left) served as the experimental units.

### 2.5 Behavioral Studies

#### 2.5.1 Spatial Recognition Memory in the Two-chamber Apparatus

The 2-chamber apparatus was used in 3 months old rats (SET1: CONT-F: n = 5, PRS-F: n = 5, CONT-M: n = 5, PRS-M: n = 7) for the assessment of spatial recognition memory, as it evaluates the capacity to distinguish between a novel and a familiar environment [45, 46]. The apparatus (IMETRONIC, Bordeaux, France) The apparatus (IMETRONIC, Bordeaux, France) was located in an experimental room separate from the housing room and consisted of two identical compartments named Z1 and Z2 (30x30x10 cm) connected by an alley named Z3 (30x10 cm large, 45 cm high) with two opposite openings, one per compartment, which could be closed by sliding doors. Boxes were covered to isolate visual cues, and the floor had photoelectric beams to track compartment time. Two spatial configurations were created using Plexiglas parallelepipeds. In the “acquisition configuration”, both compartments remained unchanged; in the “restitution configuration”, a parallelepiped was placed in one compartment (novel environment). The task comprised two trials separated by a 4-hour interval, with male and female rats tested in separate daily sessions to avoid confounding factors related to sex-specific odors. In the acquisition phase, conducted during the light cycle (between 9:00 AM and 11:00 AM), rats explored the acquisition configuration for 10 minutes. During the second trial, conducted during the light cycle but 4 hours after the intertrial interval (between 1:00 PM and 3:00 PM), they explored the restitution configuration for 15 minutes. Recognition score during the restitution trial was calculated as the percentage ratio of time spent (0-5 min) in the Z1 novel *vs.* time recorded in the Z2 familiar compartment; a score above 100% indicates spatial recognition. Entry numbers into the novel and familiar compartments were analyzed as measures of exploration.

#### 2.5.2 Exploration in the Elevated Plus Maze (EPM) Test

The EPM was used in 4-month-old rats (SET2: CONT-F: n = 6, PRS-F: n = 6, CONT-M n = 7, PRS-M n = 6). The EPM is widely used for the evaluation of anxiolytic drugs [47, 48] and, in the absence of pharmacological treatments, the number of entries and the time spent in the open arm of the EPM is a measure of risk-taking behavior in the response to novelty and adverse environment [16, 29, 30, 47, 49]. Moreover, the EPM incorporates a cognitive component (decision-making), which drives the preference to enter one of the two arms of the maze [50]. In this study, animals were submitted to one trial EPM [29]. The EPM test was performed in an experimental room separate from the housing room for 5 min early in the afternoon (between 1 and 4 pm) and began with the placement of the rat in the center of the maze with the head facing a closed arm. The test was conducted at this specific time for all animals to ensure consistency, with male and female rats tested in separate daily sessions to avoid confounding factors related to sex-specific odors. The closed arms’ luminosity was approximately 25 lx, and the luminosity of the open arms was approximately 50 lx. Behavior was recorded by a video camera and manually scored by a trained observer blind to the animals’ condition (PRS and CONT) and sex (males *vs.* females) using a software package (The Observer^®^ Noldus). We measured the time spent in the open and closed arms, the latency to enter the open arms, as well as the entries in the open and closed arms. The time spent in the open arms was considered as risk-taking behavior in an open space [51]. The discrimination between open and closed arms reflects the decision-making of the animal with respect to the threatening (open) arms or reassuring (closed) arms.

### 2.6 Western Blot Analysis

Biochemical analyses were conducted on 3-month-old animals (Set 3). Brain tissues were rapidly dissected and immediately stored at -80°C. Whenever possible, left and right tissue from the same region were collected from each animal. This allowed us to analyze the prefrontal cortex (right and left; n = 40 samples), as well as the dorsal and ventral hippocampus (right and left; n = 38 samples and n = 46 samples, respectively). Glutamate AMPA receptors and GABA_A_ receptor subunits were assessed in crude synaptosomal fraction. To isolate synaptosomes, tissue was manually homogenized with a potter in ten volumes of HEPES-buffered sucrose (0.32 M sucrose, 4 mM HEPES pH 7.4). All procedures were performed at 4°C. Homogenates were centrifuged at 1000 × g for 10 min, and the resulting supernatants were centrifuged at 10,000 × g for 15 min. The pellet obtained from the second centrifugation was resuspended in ten volumes of HEPES-buffered sucrose [25]. This pellet contained the crude synaptosomal fraction. BCA assay was used to determine protein concentration. Synaptosome lysates were resuspended in Laemmli reducing buffer, and 20 µg for a synaptosomal fraction of each sample were loaded. As more gels needed to be carried for the same marker taking into account the number of lanes available, to ensure consistency across gels and to facilitate comparison of samples between different gels, one of the samples was used as an internal control and loaded onto each gel. Proteins were first separated by electrophoresis on sodium dodecyl sulfate-polyacrylamide gels according to their molecular weight and then transferred to nitrocellulose membranes (Bio-Rad). The transfer was performed at 4°C in a buffer containing 35 mM Tris, 192 mM glycine, and 20% methanol. After transfer, blots were incubated in a blocking solution containing Tris-buffered saline and 5% (w/v) non-fat milk. All the following antibodies were first tested with control samples to determine the optimal conditions for use. To analyze several proteins per membrane, membranes were cut according to the molecular weight of the protein of interest. We used the following primary antibodies on crude synaptosomal fraction: rabbit monoclonal anti-GluA_1_ (1:2000; catalog #ab31232; Abcam), rabbit monoclonal anti-GluA_2_ (1/2000; catalog #ab206293), mouse monoclonal anti-GluA_3_ (1/800; catalog #MAB5416) purchased from Merck; GABA_A_ subunits: rabbit polyclonal anti-GABA_A_ receptor α_1_ (1:1000. catalog #06-868, Sigma-Aldrich); rabbit polyclonal anti-GABA_A_ receptor α_2_ (1:1500; catalog #ab72445), rabbit polyclonal anti-GABA_A_ receptor α_4_ (1:1000; catalog #AB5459), rabbit polyclonal anti-GABA_A_ receptor γ_2_ (1:500; catalog #G0545) purchased from Merck; rabbit polyclonal anti-GABA_A_ receptor δ (1:800; catalog #NBP3-03679) purchased by from Novus Biological; GABA cotransporters: rabbit polyclonal anti-KCC2 (1:5000 catalog #07-432, Sigma Aldrich), rabbit polyclonal anti-NKCC1 (1:800; catalog #13884-1-AP) purchased from Proteintech. To ensure that each lane was loaded with an equivalent amount of proteins, the blots were probed with a mouse monoclonal anti-b-actin (1:1500; catalog #A5316, Sigma). All primary antibodies were incubated overnight at 4°C. Horseradish peroxidase-conjugated secondary anti-mouse or anti-rabbit antibodies (purchased from GE-Healthcare) were used at a dilution of 1:5000 and incubated for 1 h at room temperature. Bands were visualized with an enhanced chemiluminescence system (ECL enhancer Thermofisher). After immunoblotting, digitized images of bands immunoreactive for target antibodies and actin were acquired (FUSION^®^), and the area of immunoreactivity corresponding to each band (O.D., optical density) was measured using ImageJ. A ratio of target to actin was then determined and data were expressed as a percentage of the control female group. Moreover, we used the receptor subunit protein densities, calculated as described above, to determine the ratio between AMPA and GABA receptor subunits. This ratio was calculated by dividing the sum of the target-to-actin ratios, expressed as a percentage of the control female group, for the AMPAR subunits by the sum of the target-to-actin ratios for the GABA_A_R [52].

### 2.7 Statistical Analysis

STATISTICA 8.0 (StatSoft, Inc.) was used for power analysis (power goal set at 0.8), sample size calculation, and data analysis. Behavioral and biochemical data were analyzed using a parametric analysis of variance (ANOVA) with Group-Stress (CONT *vs.* PRS) and Sex (male *vs.* female) as independent variables. Results are expressed as the mean ± standard error of the mean (S.E.M.). Post-hoc comparisons were performed using the Newman-Keuls (NK) test and confirmed with Bonferroni’s test. Outliers in immunoblotting data were identified using the Grubbs test and removed if Grubbs’s G > 0.05 [53]. For principal components analysis (PCA), PERMANOVA [54] was used to test group differences and effects of variables in a multivariate manner. A *p*-value of <0.05 was considered statistically significant. All graphs were created with GraphPad PRISM Version 10.2.3 (MA, USA).

## 3 RESULTS

### 3.1 Sex-Specific Impact of PRS on Spatial Recognition Memory and Risk-Taking Behavior in Adult Rats

**Fig. (2):**
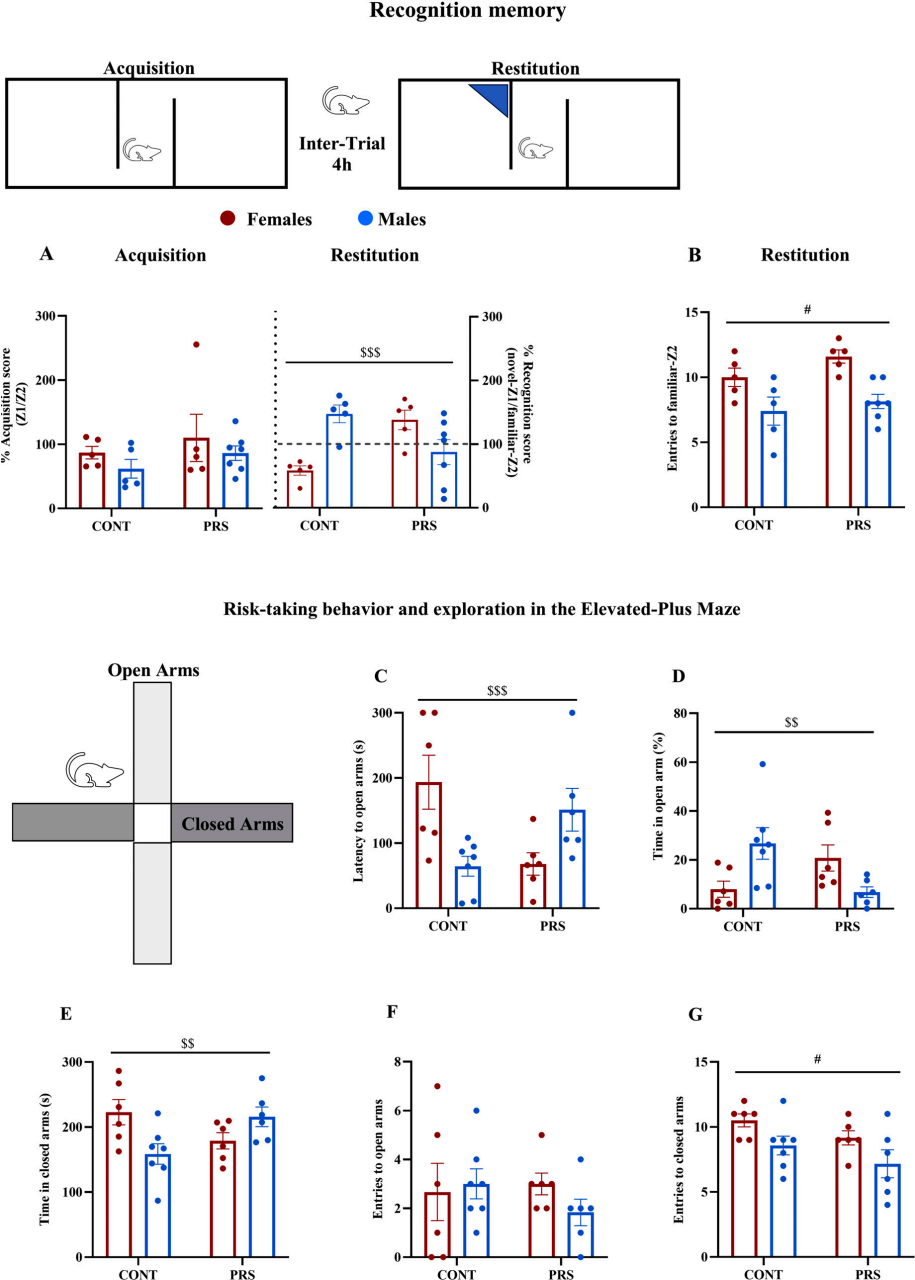
Sex-specific effect of PRS on spatial recognition memory in the two-chamber apparatus and risk-taking behavior and exploration in the Elevated-Plus Maze (EPM) of adult rats. Adult PRS and control rats of both sexes were assessed for spatial recognition memory using the 2-chamber apparatus (**A**, **B**) and for risk-taking behavior in the elevated plus maze (**C-G**). Values are expressed as means ± S.E.M. (n = 5-7 rats/group); *main sex effect*: #= *p* <0.05; *PRS x sex interaction*: $$= *p* <0.01; $$$= *p* <0.001.

During the acquisition trial on the 2-chamber test, there were no differences between the four groups (Fig. **2A**, Acquisition). We assessed spatial recognition memory by quantifying in a two-chamber apparatus the recognition score of the novel environment-Z1, which represents the percentage of time spent in the novel compartment as a function of the recognition spatial memory score during the restitution trial. Notably, no main effect of sex nor PRS was observed, while the impact of PRS was found to be sex-dependent (*PRS × sex interaction*, n = 5-7 rats/group, F_(1,18)_= 18.1916, *p* = 0.00047), as the recognition score significantly decreased in PRS-exposed males and increased in PRS-exposed females, when compared to their respective control groups (post-hoc analysis: PRS-M *vs.* CONT-M, *p* = 0.046; PRS-F *vs.* CONT-F, *p* = 0.0079) (Fig. **2A**, Restitution). Also, there were sex differences in the recognition score between male and female PRS and within the male and female control groups (post-hoc analysis: PRS-M *vs.* PRS-F, *p* = 0.042; CONT-M *vs.* CONT-F, *p* = 0.006). A main effect of sex was observed for the entries to the familiar-Z2 environment, which were increased in females with respect to males independently of the PRS (*sex effect*, F_(1,18)_ = 17.344, *p* = 0.00058) (Fig. **2B**, Restitution).

Adult rats exposed to PRS and their respective control counterparts, encompassing both sexes, underwent an assessment of risk-taking behavior in the open and new arms of EPM by measuring the latency to enter the open arm (Fig. **2C**), the time spent in the open (Fig. **2D**), and closed arms (Fig. **2E**), and the number of entries to the open (Fig. **2F**), and closed arms (Fig. **2G**).

No main effect of sex nor PRS was observed, while the latency to enter in the open arm was found to be increased in control females compared to control males, and PRS reversed sex difference observed in controls with PRS females displaying reduced latency to enter in the open arm with respect to PRS males (n = 6-7 rats/group, latency to open arm: *PRS × sex interaction*, F_(1,21)_=14.39, *p =* 0.0010; post-hoc analysis: CONT-M *vs.* CONT-F, *p =* 0.018; PRS-M *vs.* PRS-F, *p =* 0.048; post-hoc analysis: PRS-F *vs*. CONT-F, *p =* 0.012). Differently, PRS males showed the same latency as control males (post-hoc analysis: PRS-M *vs*. CONT-M, *p =* 0.096) (Fig. **2C**). Also, the time spent in the open arms was different between the four groups (n = 6-7 rats/group, time in the open arms: *PRS × sex interactio*n, F_(1,21)_=11.43, *p =* 0.00281) (Fig. **2D**), without the main effect of sex, not PRS. In particular, sex differences were observed in the controls (post-hoc analysis: CONT-M *vs.* CONT-F, *p =* 0.032) and not in PRS groups (post-hoc analysis: PRS-M *vs.* PRS-F, *p =* 0.126). Moreover, male rats subjected to PRS showed a reduced time spent in the open arms in comparison to their unstressed control counterparts (post-hoc analysis: PRS-M *vs*. CONT-M, *p =* 0.038), while there was no difference between PRS and control females (post-hoc analysis: PRS-F *vs*. CONT-F, *p =* 0.076) (Fig. **2D**). The time spent in the closed arms (Fig. **2E**) was opposite to the time spent in the open arms, and a different profile was observed within the four groups (n = 6-7 rats/group, *PRS × sex interaction*, F_(1,21)_=9.989, *p =* 0.0047) in absence of main effect of sex or PRS. In particular, a sex difference in the control groups revealed that females spent more time than males in the closed arms (post-hoc analysis: CONT-M *vs.* CONT-F, *p =* 0.045) and a PRS effect within male groups indicated that PRS males spent more time in the closed arms than controls (post-hoc analysis: PRS-M *vs*. CONT-M, *p =* 0.049). No effect of sex or PRS was observed when measuring the number of entries in the open arm (Fig. **2F**). Conversely, when measuring the number of entries in the closed arm (Fig. **2G**), as a measure of activity, a main effect of sex revealed that females were more reactive than males regardless of PRS (n = 6-7 rats/group, *sex effect*, F_(1,21)_=6.918, *p =* 0.0156).

### 3.2 Sex- and Brain Region-Specific Effects of PRS on AMPA Receptor Subunits, GABA_A_ Receptor Subunits, and Membrane Cl^-^ Transporters

We measured protein levels of GluA1, GluA2, and GluA3 AMPA receptor subunits, α_1_, α_2_, α_4_, γ2 and δ GABA_A_ receptor subunits, and the Cl^-^ transporters, NKCC1 and KCC2, by immunoblotting. Moreover, we analyzed the ratio of protein optical densities normalized to β-actin for GluA2 and GluA1 (GluA2:GluA1 ratio), for KCC2 and NKCC1 (NKCC1:KCC2 ratio) and for the overall sum of AMPAR and GABA (AMPAR:GABA_A_R ratio subunits). These ratios were analyzed in relation to the presence or absence of the GluA2 subunit, which regulates calcium permeability, as well as the synaptic (α_1_, α_2_, γ2) or extra-synaptic (α_4_, δ) localization of GABA_A_R receptor subunits. For each selected protein, Western blots showed a single band at the expected molecular size (see blots in supplementary Figures).

### 3.3 Prefrontal Cortex

**Fig. (3):**
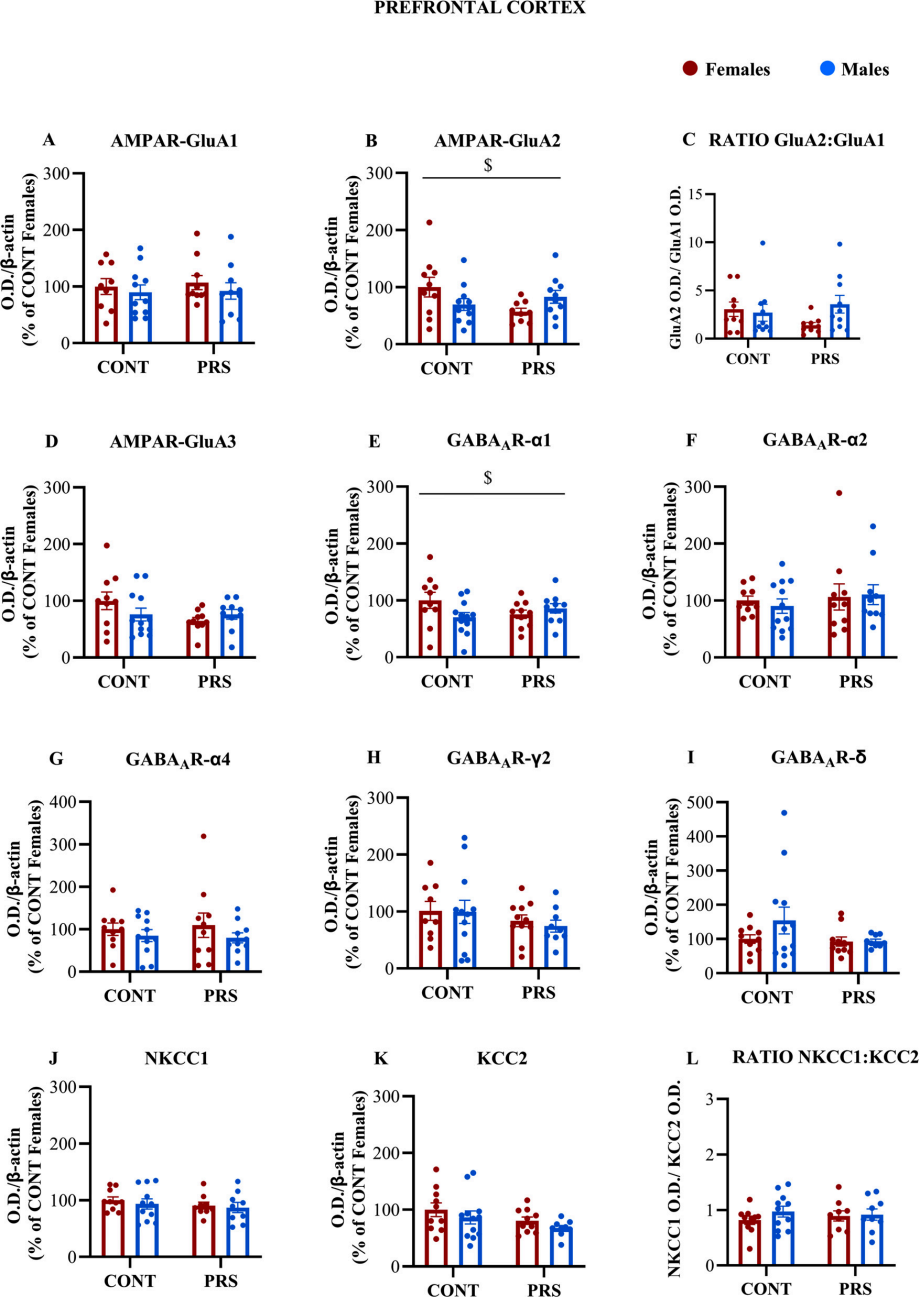
AMPAR, GABA_A_ expression, and GABA_A_ receptor cationic transporters KCC2 and NKCC1 expression in the prefrontal cortex of adult rats. Immunoblot analysis of AMPAR GluA1, GluA2, and GluA3 subunits and the resulting GluA_2_:GluA_1_ ratio in crude synaptosomal fractions in PRS and CONT unstressed rats of both sexes. (**A-D**). Immunoblot analysis of GABA_A_ receptor α1, α2, α4, δ, and γ2 subunits (**E-I**) and NKCC1, KCC2, and their resulting NKCC1: KCC2 ratio (**J-L**) in the same synaptosomal fraction of groups above reported. Values are expressed as means ± S.E.M. (n = 9-12 PFC tissue/group, adults); *PRS x sex interaction*: $= *p* <0.05. O.D.=optical density.

Results are shown in Fig. (**3**). There was no discernible effect of PRS or sex on GluA1 (Fig. **3A**) and GluA3 (Fig. **3D**) protein levels in the prefrontal cortex (PFC). In contrast, PRS induced sex-dependent changes in the levels of the Ca^2+^-impermeable GluA2 subunit. Specifically, in the absence of the main effect of sex or PRS, GluA2 protein levels (Fig. **3B**) were different between the four groups (one outlier in CONT-M and one in PRS-F were identified by Grubbs test in GluA2 and therefore removed; n = 9-11 PFC/group, CONT-F=10; PRS-F=9; CONT-M=8+3 internal standards; PRS-M=10; *PRS × sex interaction*, F_(1,36)_=5.501; *p =* 0.0246), although post-hoc analysis did not reveal any significant difference comparing PRS and sex. One outlier in CONT-F and one in CONT-M were identified by the Grubbs test in GluA1 and therefore removed. Concerning the GluA2:GluA1 ratio (Fig. **3C**), no main effect of sex nor PRS was observed when comparing the 4 groups (GluA2:GluA1 ratio: CONT-F=9; PRS-F=9; CONT-M=7+3 internal standards; PRS-M=10; n = 9-10 PFC/group, *PRS × sex interaction*, F_(1,34)_=2.68; *p =* 0.110). GABA_A_ receptor subunits and Cl^-^ transporters were unaffected by sex and PRS in the prefrontal cortex (Fig. **3F**-**K**), with the exception of the α_1_ subunit where a PRS x sex interaction revealed that α_1_ protein levels were different between the four groups (n = 10-12 PFC/group, CONT-F=10; PRS-F=10; CONT-M=9+3internal standards; PRS-M=10; *PRS x sex interaction* F_(1,38)_=4.144, *p =* 0.048), although post-hoc analysis did not reveal any significant difference comparing PRS and sex (Fig. **3E**).

### 3.4 Dorsal Hippocampus

**Fig. (4):**
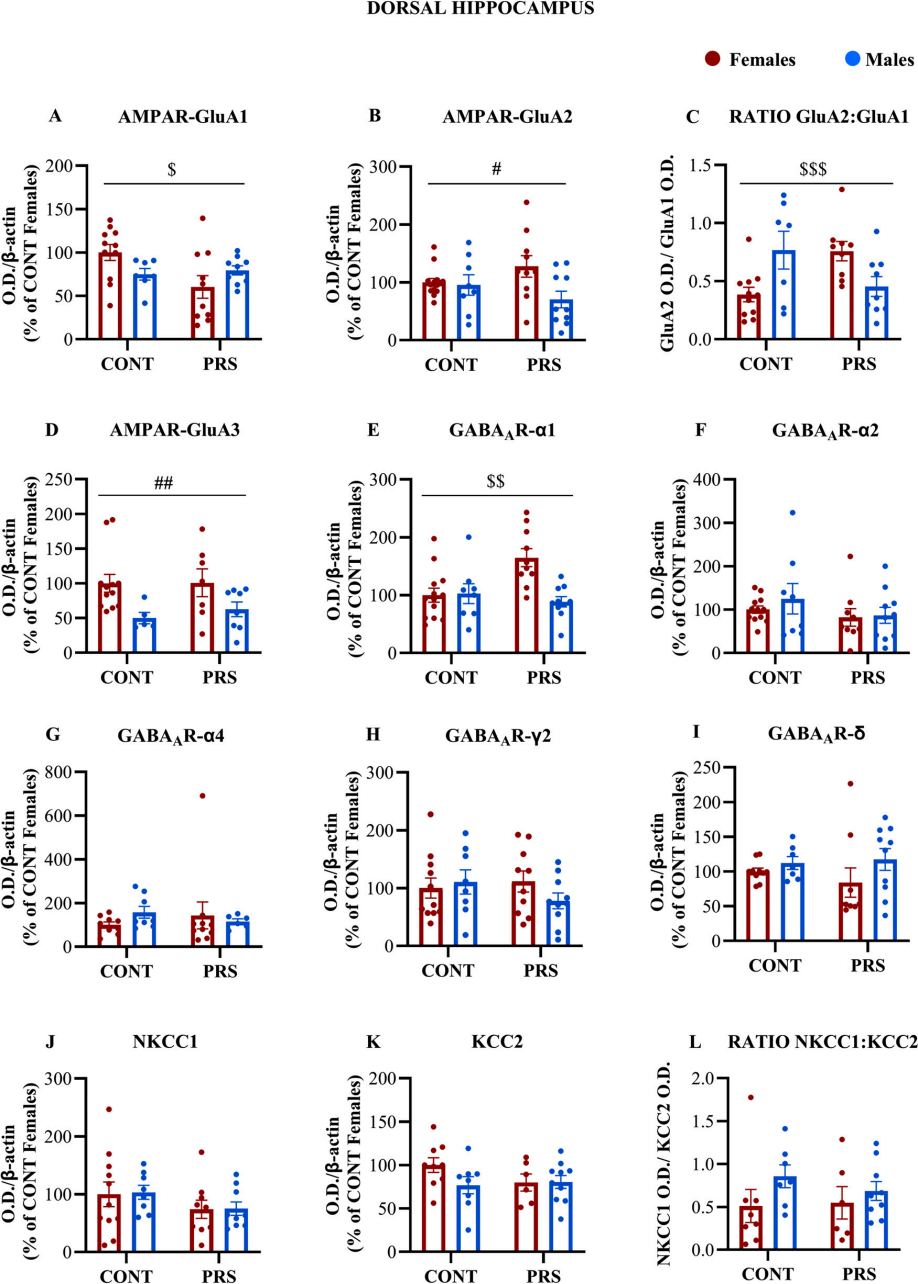
AMPAR, GABA-_A_ receptor subunits, and GABA_A_ receptor cationic transporters KCC2 and NKCC1 expression in the dorsal hippocampus of adult rats. Immunoblot analysis of AMPAR GluA1, GluA2, and GluA3 subunits and the resulting GluA2:GluA1 ratio in crude synaptosomal fractions in PRS and CONT unstressed rats of both sexes. (**A-D**). Immunoblot analysis of GABA_A_ receptor α1, α2, α4, γ2 and δ subunits (**E-I**) and NKCC1, KCC2, and their resulting NKCC1:KCC2 ratio (**J-L**) in the same synaptosomal fraction of groups above reported. Values are expressed as means ± S.E.M. (n = 5-14 HPC-D/group); main *sex effect*: #= *p* <0.05; ##= *p*< 0.01; *PRS x sex interaction*: $= *p* <0.05, $$= *p* <0.01, $$$= *p* <0.001; O.D.=optical density.

In the dorsal hippocampus, no main effect of sex or PRS was observed for GluA1 (Fig. **4A**) which was different between the four groups (one outlier in CONT-F, one in CONT-M, and one in PRS-M were identified by Grubbs test in GluA1 and therefore removed. n = 7-11 HPC-D/group, CONT-F=8+3 internal standards; PRS-F=10; CONT-M=7; PRS-M=9; *PRS x sex interaction*, F_(1,33)_=5.041, *p =* 0.03157), however post-hoc analysis revealed only that PRS significantly reduced GluA1 levels in females compared to control females (post-hoc analysis: PRS-F *vs.* CONT-F, *p =* 0.035). Conversely, GluA2 protein levels (Fig. **4B**) were diminished in males compared to females, irrespective of PRS exposure (n = 8-14 HPC-D/group, CONT-F=9+5 internal standards; PRS-F=10; CONT-M=8; PRS-M=10; *main sex effect*, F(_1,38)_=4.912; *p =* 0.0327). Furthermore, PRS led to an increase in the GluA2:GluA1 ratio (Fig. **4C**) in females and a reduction in males, with opposite effects observed in unstressed control rats and without the main effect of PRS or sex (one outlier in PRS-F was identified by Grubbs test for the GluA2:GluA1 ratio and therefore removed. n = 7-11 HPC-D/group, CONT-F=8+3 internal standards; PRS-F=9; CONT-M=7; PRS-M=9; *PRS x sex interaction*, F_(1,32)_= 12.995; *p =* 0.0010; post-hoc analysis: CONT-M *vs.* CONT-F, *p =* 0.037; PRS-M *vs.* PRS-F, *p =* 0.031; PRS females *vs.* CONT females *p =* 0.024; PRS males *vs.* CONT males *p =* 0.066). Lastly, GluA3 protein levels (Fig. **4D**) exhibited sex-dependent differences irrespective of PRS exposure, with higher levels observed in females (one outlier in PRS-F, and one in CONT-M were identified by Grubbs test for GluA3 and therefore removed. n = 5-12 HPC-D/group, CONT-F=9+3 internal standards; PRS-F=7; CONT-M=5; PRS-M=8; *main sex effect*, F_(1,28)_=8.592; *p =* 0.00665). Similarly, to what was observed in the prefrontal cortex, the levels of GABA_A_ receptor subunits and Cl^-^ transporters in the dorsal hippocampus were minimally affected by sex and PRS exposure. PRS exposure led to increased α1 protein levels (Fig. **4E**) in females compared to control females and PRS males, while no significant change was observed in PRS males compared to control groups (one outlier in CONT-F was identified by Grubbs test for α1 subunit and therefore removed. n = 8-13 HPC-D/group, CONT-F=9+4 internal standards; PRS-F=10; CONT-M=8; PRS-M=10; *main sex effect*, F_(1,37)_=7.340; *p =* 0.01; *PRS × sex interaction*, F_(1,37)_=8.416; *p =* 0.00622; post-hoc analysis: PRS-F *vs.* CONT-F, *p =* 0.005; PRS-M *vs.* PRS-F, *p =* 0.0019).

Levels of α_2_, α_4_, γ_2_, and δ subunits, and levels of KCC2 and NKCC1 were not affected by sex nor PRS exposure (Figs. **4F**-**L**).

### 3.5 Ventral Hippocampus

**Fig. (5):**
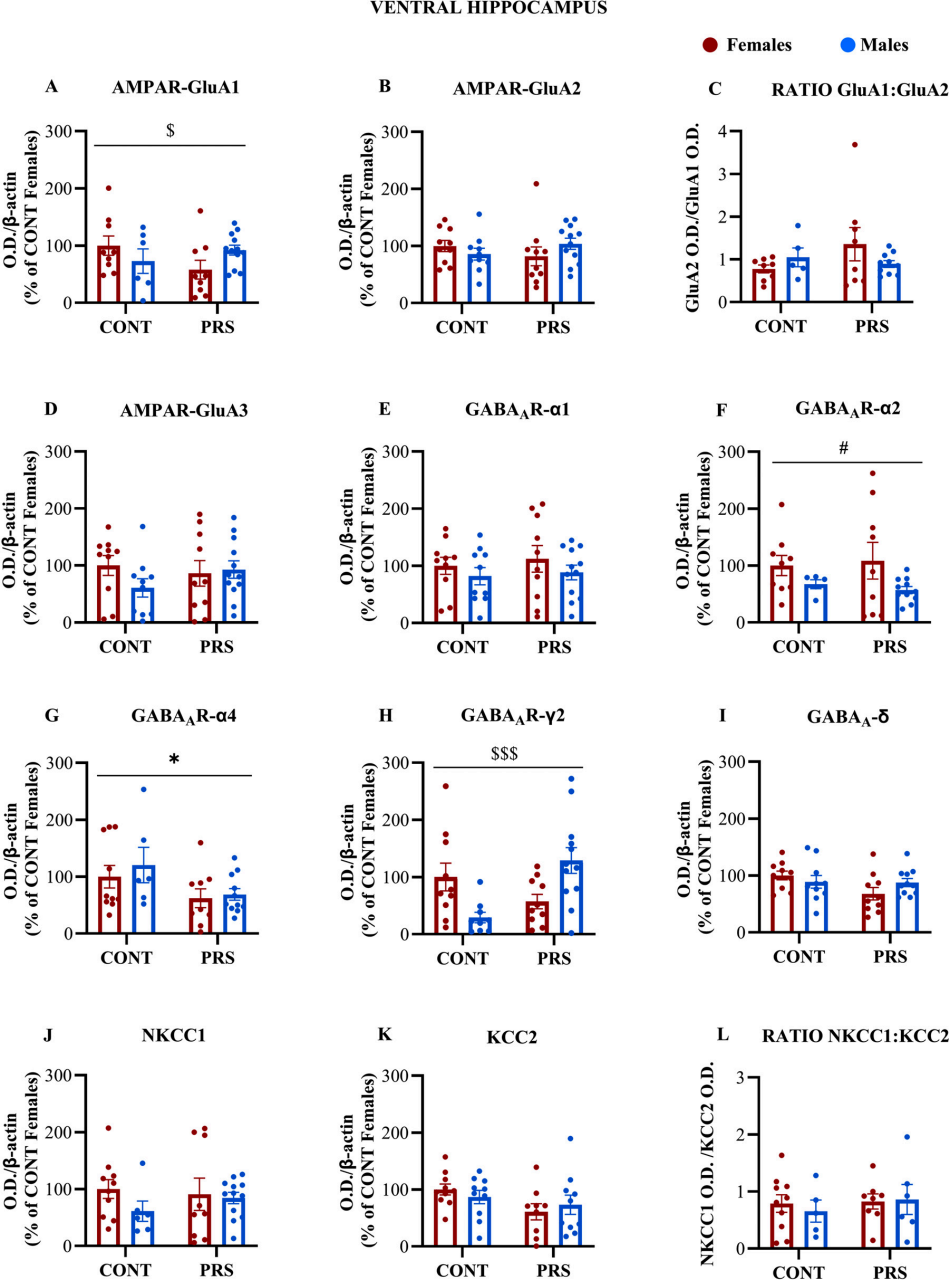
AMPAR and GABA_A_ receptor subunits and GABA_A_ receptor cationic transporters KCC2 and NKCC1 expression in the ventral hippocampus of adult rats. Immunoblot analysis of AMPAR GluA1, GluA2, and GluA3 subunits and the resulting GluA2:GluA1 ratio in crude synaptosomal fractions in PRS and CONT unstressed rats of both sexes. (**A-D**). Immunoblot analysis of GABA_A_ receptor α1, α2, α4, γ2 and δ subunits (**E-I**) and NKCC1, KCC2, and their resulting NKCC1: KCC2 ratio (**J-L**) in the same synaptosomal fraction of groups above reported. Values are expressed as means ± S.E.M. (n = 5-12 HPC-V/group); *main PRS effect*: * = *p* <0.05; *main sex effect:* # = *p* <0.05; *PRS x sex interaction*: $= *p* <0.05, $$$= *p* <0.001. O.D.=optical density.

Results are shown in Fig. (**5**). Notably, we did not observe significant alterations in the levels of AMPA receptor subunits within the ventral hippocampus among the four groups of rats (Figs. **5A**-**D**). However, in the absence of a main effect of PRS or sex, a *PRS x sex interaction* was observed when analyzing the GluA1 subunit (one outlier in CONT-F was identified by Grubbs test for GluA1; n = 6-12 HPC-V/group; CONT-F=9; PRS-F=9; CONT-M=6; PRS-M=9+3 internal standards. *PRS x sex interaction* F_(1,32)_= 4.0439; *p =* 0.0528) (Fig. **5A**), although the specific post-hoc comparisons showed no differences between the PRS and control groups for either females or males (Fig. **5A**). Despite the profile of GluA1 expression levels, we did not observe statistically significant changes in the GluA2:GluA1 ratio (Fig. **5C**).

In contrast, the expression levels of the α_2,_ α_4_, and γ_2_ subunits of GABA_A_ receptors in the ventral hippocampus were influenced by sex and/or PRS exposure. The level of the α_2_ subunit was found to be higher in females regardless of their group (one outlier in CONT-F, one in CONT-M and one in PRS-M were identified by Grubbs test and removed; n = 5-11 HPC-V/group; CONT-F=9; PRS-F=9; CONT-M=5; PRS-M=8+3 internal standards. *main sex effect*, F_(1,30)_=4.0423; *p =* 0.053) (Fig. **5F**). The levels of the α_4_ subunit were found to be lower in PRS-exposed rats regardless of their sex (one outlier in PRS-M was identified by Grubbs test and removed; n = 6-11 HPC-V/group; CONT-F=10; PRS-F=9; CONT-M=6; PRS-M=8+3 internal standards. main *PRS effect*, F_(1,32)_=5.750; *p =* 0.022) (Fig. **5G**). Regarding the γ_2_ subunit, in absence of a main effect of PRS or sex, a *PRS x sex interaction* revealed divergent expression profiles in control and PRS-exposed rats (One outlier in CONT-M as identified by Grubbs test and removed; n = 9-12 HPC-V/group; CONT-F=10; PRS-F=10; CONT-M=9; PRS-M=8+3 internal standards. *PRS x sex interaction* F_(1,37)_=13.492; *p =* 0.0007). Specifically, levels were lower in control males compared to control females (post-hoc analysis: CONT-M *vs.* CONT-F, *p =* 0.036), higher in PRS-exposed males compared to PRS-exposed females (post-hoc analysis: PRS-M *vs.* PRS-F, *p =* 0.034), and higher in PRS-exposed males compared to control males (post-hoc analysis: PRS males *vs.* CONT males, *p =* 0.0046). However, there was no difference in γ2 subunit levels between PRS and control females (post-hoc analysis: PRS-F *vs.* CONT-F, *p =* 0.127) (Fig. **5H**). No significant alterations were observed in the α_1_ and δ GABA_A_ receptor subunits, as well as in KCC2 and NKCC1 protein levels (Figs. **5E**, **I**-**L**).

### 3.6 AMPA and GABA_A_ Receptor Subunits Ratio

**Fig. (6):**
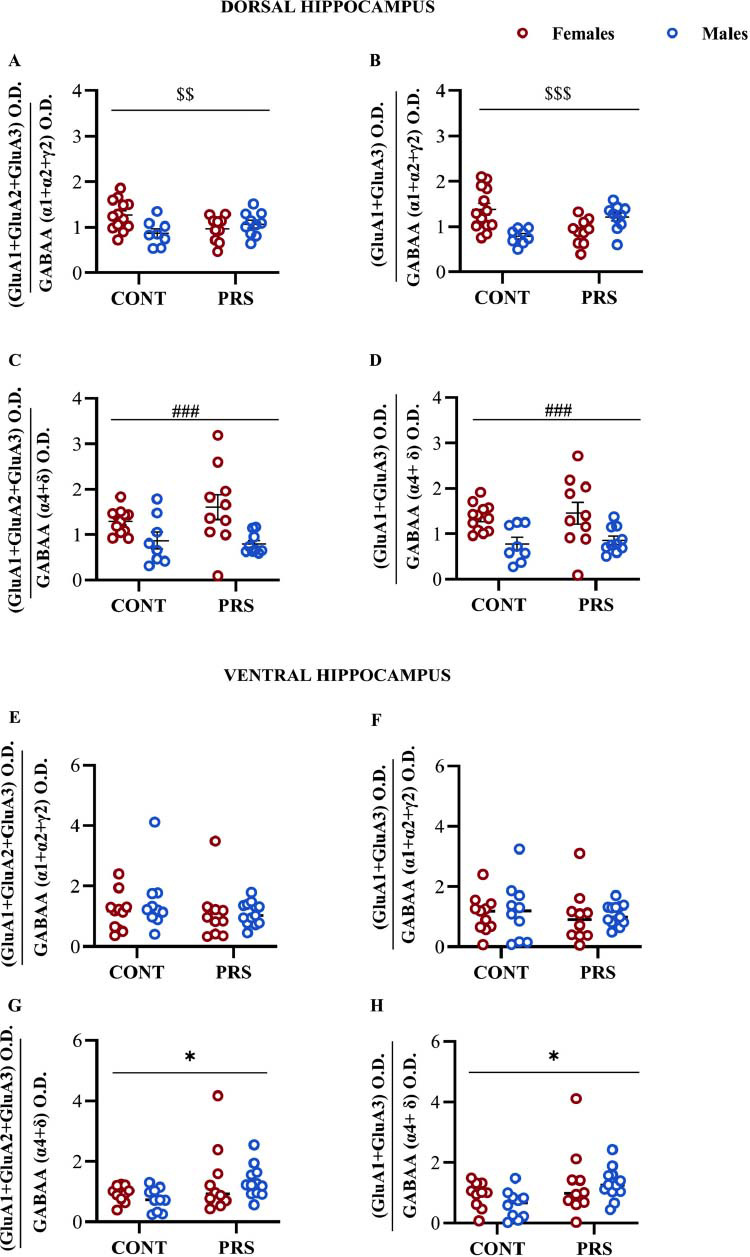
AMPA and GABA_A_ Receptors Ratio in the dorsal and ventral hippocampus of adult rats. AMPAR:GABA_A_R ratios in PRS and CONT unstressed rats of both sexes, considering both the presence and absence of the GluA2 subunit and the synaptic (α_1,_ α_2,_ γ2) or extra-synaptic (α_4,_ δ) localization of GABA_A_ receptors subunits in the dorsal hippocampus (**A-D**) and ventral hippocampus (**E-H**). Values are expressed as means ± S.E.M. (n = 8-13 HPC-D or HPC-V/group); *main PRS effect*: * = *p* <0.05; *main sex effect*: ### = *p* <0.001; *PRS x sex interaction*: $$= *p* <0.01, $$$= *p* <0.001. O.D.=optical density.

We observed no significant changes in synaptic and extra-synaptic AMPAR:GABA_A_R ratios in the prefrontal cortex across the four groups of rats analyzed (data not shown). Conversely, a significant modulation of the AMPAR: GABA_A_R ratio was found in the dorsal hippocampus at both synaptic and extra-synaptic levels, with and without the GluA2 subunit (Figs. **6A**-**D**). Specifically, when summing AMPA subunits containing GluA2 relative to synaptic GABAergic subunits (α1, α2, and γ2, Fig. **6A**), we observed a significant *PRS × sex interaction* without main effects (n = 8-13 HPC-D/group; CONT-F=13; PRS-F=10; CONT-M=8; PRS-M=10. *sex × PRS interaction*, F_(1,37)_=7.354, *p =* 0.01). However, only control males showed lower levels compared to control females (post-hoc analysis: CONT-M *vs.* CONT-F, *p =* 0.02), indicating a predominance of inhibition over excitation in the control male group compared to females. When the AMPAR:GABA_A_R ratio was calculated without the GluA2 subunit (Fig. **6B**), more pronounced differences emerged between the four groups, with a clear *PRS × sex interaction* (n = 8-13 HPC-D/group; CONT-F=13; PRS-F=10; CONT-M=8; PRS-M=10, *sex × PRS interaction*, F_(1,37)_=18.429, *p =* 0.0001) that revealed divergent expression profiles in PRS-exposed rats compared to controls without any main effects. Specifically, control males had lower AMPAR:GABA_A_R ratio levels than control females (post-hoc analysis: CONT-M *vs.* CONT-F, *p =* 0.0019), PRS-exposed males had higher levels than PRS-exposed females (post-hoc analysis: PRS-M *vs.* PRS-F, *p =* 0.04), PRS-exposed males had higher levels than control males (post-hoc analysis: PRS-M *vs*. CONT-M, *p =* 0.02), while PRS females had reduced levels than control females (post-hoc analysis: PRS-F *vs*. CONT-F, *p =* 0.007) indicating that control males and PRS females are more inhibited than the other 2 groups. While considering extra-synaptic GABAergic subunits (α4, δ) in the AMPAR:GABA_A_R ratio within the dorsal hippocampus (Figs. **6C**, **D**), a significant main effect of sex was observed, both with GluA2 (Fig. **6C**: n = 8-12 HPC-D/group; CONT-F=12; PRS-F=10; CONT-M=8; PRS-M=10, *sex effect*, F_(1,36)_=14.114, *p =* 0.0006) and without the GluA2 subunit (Fig. **6D**: n = 8-12 HPC-D/group; CONT-F=12; PRS-F=10; CONT-M=8; PRS-M=10, *main sex effect*, F_(1,36)_=15.160, *p =* 0.0004), with females showing increased excitation relative to inhibition compared to males, regardless of the group. Concerning the ventral hippocampus, no significant differences were observed when the ratio AMPAR:GABA_A_R subunits included only synaptic GABAergic subunits (Figs. **6E**, **F**). In contrast, when extra-synaptic GABAergic subunits were considered in the excitation/inhibition balance analysis of the ventral hippocampus (Figs. **6G**, **H**), we observed that the AMPAR-to-GABA_A_R ratio was affected by PRS exposure, independently of the sex. Specifically, in PRS-exposed rats of both sexes, the AMPAR:GABA_A_R ratio was higher compared to controls, both in the presence of GluA2 (Fig. **6G**: n = 10-12 HPC-V/group; CONT-F=10; PRS-F=10; CONT-M=10; PRS-M=12, *main PRS effect*, F_(1,38)_=6.084; *p =* 0.018) and absence of the GluA2 subunit (Fig. **6H**: n = 10-12 HPC-V/group; CONT-F=10; PRS-F=10; CONT-M=10; PRS-M=12, *main PRS effect*, F_(1,38)_=6.288; *p =* 0.016).

### 3.7 PCA Multidimensional Analysis

As shown in Fig. (**7**), in the dorsal hippocampus, PERMANOVA analysis unveiled a *PRS x sex interaction* (*p =* 0.050), with notable distinctions primarily within the female PRS group, which displayed a clustering phenomenon distinct from other groups (PRS-F *vs*. CONT-F, *p =* 0.062 and PRS-F *vs*. PRS-M: p = 0.053). Conversely, in the ventral hippocampus, a *PRS x sex interaction* (*p =* 0.046) highlighted significant differences predominantly within the male groups, where PRS males exhibited closer proximity to both PRS and control female groups than to control males (PRS-M *vs.* CONT-M, *p =* 0.024). Noteworthy, in the prefrontal cortex, no discernible differences were observed across the groups (*sex x PRS interaction, p* = 0.36, N.S).

## 4 DISCUSSION

Our study on a preclinical model of ELS presents compelling evidence that PRS induces sex-dependent modifications in behavioral response to novelty and cognitive processes that are associated with the specific region-alterations in the subunit expression of AMPA and GABA_A_ receptors, the two ionotropic receptors responsible for mediating fast excitatory and inhibitory neurotransmission. These findings provide valuable insights into the neurochemical framework underlying PRS effects, highlighting the excitatory/inhibitory balance mediated by AMPA and GABA_A_ receptors as a potential therapeutic target for neuroplasticity dysfunctions. Although the translational relevance of these results to humans requires careful consideration, given species-specific differences in neurodevelopmental trajectories, receptor expression patterns, and behavioral manifestations of stress, our data sustain the importance of developing gender-specific therapeutic approaches to effectively address the disorders associated with ELS.

### 4.1 PRS and Sex Effects on AMPA and GABA_A_ Receptors in the Dorsal Hippocampus and Prefrontal Cortex

PRS led to sex-dependent changes in the GluA2:GluA1 ratio of AMPAR and in α1-GABA_A_ levels in the dorsal hippocampus and prefrontal cortex, two main regions involved in recognition memory [55]. The GluA2:GluA1 ratio may reflect the functional status of excitatory synapses. Indeed, the GluA2 subunit restrains Ca^2+^ permeability of the AMPA-gated ion channel, and surface expression of GluA2-lacking AMPA receptors is enhanced during long-term potentiation (LTP) of excitatory synaptic transmission [56-58]. Therefore, an increased efficacy of synaptic transmission results from a decreased GluA2:GluA1 ratio. However, the increased GluA2:GluA1 ratio may lower the background noise, thereby enhancing the signal-to-noise ratio during learning, resulting in an improved learning performance [59].

Our results in the dorsal hippocampus show that, while the GluA2:GluA1 ratio was greater in unstressed control males than female rats, in the PRS group the GluA2:GluA1 ratio was largely enhanced in females and reduced in males. This suggests that PRS causes opposite changes in the efficacy of excitatory synaptic transmission in the dorsal hippocampus of male and female rats compared to control groups, with PRS-exposed females displaying increased GluA2: GluA1 ratio and improved cognitive performances associated with a higher risk-taking behavior. Indeed, the increased GluA2:GluA1 ratio in the dorsal hippocampus is in line with data obtained in the two-chamber test, in which the recognition score was greater in control males than in control females, while it was higher in PRS females than in PRS males. Interestingly, changes in AMPA receptor subunits in the prefrontal cortex were opposite to those found in the dorsal hippocampus, with PRS reducing the GluA2:GluA1 ratio in females. This is not consistent with the memory-enhancing effect of PRS in females if we uniformly apply the principle of the signal-to-noise ratio to all regions of the circuit involved in recognition memory [55]. Although a functional interaction between the medial prefrontal cortex and the hippocampus is necessary for recognition memory performance [60], the two regions encode different aspects of recognition memory. As opposed to the hippocampus, the medial prefrontal cortex is not involved in simple item recognition memory, although it shares with the hippocampus a key role in object-in-place associative recognition memory, as well as in temporal order and episodic memory [55]. This suggests that different synaptic tuning and networks for plasticity between the prefrontal cortex and dorsal hippocampus might coordinate the role of the two regions in the pro- and anti-cognitive effect of ELS in female and male rats, respectively. On the other hand, the effect of PRS on spatial learning in male rats has been well documented, showing a clear link between ELS and impaired spatial learning, correlated with reduced protein levels of AMPA receptor subunits, as confirmed by most research [61, 62]. In the dorsal hippocampus, we also observed elevated levels of the GABA_A_-α1 subunit of PRS females that may also contribute to reduced background synaptic noise. Although the changes in the hippocampal α1 subunit have not been extensively studied in PRS rat models, recent research has evidenced in a rat model of juvenile stress, the involvement of this subunit in the resilience mechanisms related to the hippocampal circuits [63]. Additionally, we observed that in the prefrontal cortex, the expression of the GABA_A_-α1 subunit was affected by PRS and sex. This pattern aligns with the subunit's known role in cognitive processes [64], which could be associated with improved cognitive performance [65]. Of note, the risk-taking behavior in the EPM incorporates also a cognitive component that affects decision-making during maze exploration of the closed *vs.* open arms [50]. Thus, the sex-dependent modulation of GABA_A_-α1 subunit also could potentially underlie the pro-cognitive effects of PRS observed in resilient female rats, as evidenced by their higher performance compared to PRS males in the two-chamber test and in the different decision-making observed in the EPM. These findings are consistent with previous studies showing that PRS-induced alterations of GABA_A_-α1 subunit in males can disrupt GABA_A_ receptor function, significantly impacting the maturation of GABAergic signaling in the prefrontal cortex and leading to memory impairments in several critical developmental stages [66, 67].

### 4.2 PRS and Sex Effects on AMPA and GABA_A_ Receptors in the Ventral Hippocampus

When considering AMPAergic and GABAergic transmission in the ventral hippocampus, which regulates the stress response and behavioral reactivity to novelty [68, 69], the scenario is different and we mainly observed sex-and PRS modulation specifically for AMPA-GluA1 and GABA_A_-γ2 subunits. The sex-dependent profile of AMPA-GluA1 receptor subunit in the ventral hippocampus, in both control and PRS groups, could contribute to the differential decision-making towards the adverse-novel space (open arm) observed in the EPM. Indeed, a modulation of GluA1 AMPA receptors is known to be associated with a reduced approach-avoidance-based paradigms observed in light/dark tests and in the EPM [70]. Also, benzodiazepine withdrawal increases the exploration of the open arm in the EPM that is antagonized by selective AMPA GluA1 GYKI 52466 [71].

Interestingly, we found reduced GABA_A_-γ2 levels in PRS females and enhanced in PRS males, mirroring the increased resilience in the EPM test of PRS females with respect to males. These behavioral results are consistent with previous studies performed in other prenatal stress models [72, 73]. Furthermore, the selective invalidation of the γ2 subunit specific to somatostatin (SST) neurons of the hippocampus has been proven to increase risk-taking behavior and exploration in the EPM without affecting spatial recognition memory [74]. The increase in the hippocampal γ2 subunit observed in PRS male rats is in accordance with an exacerbation of seizure-related phenotype showed in a study reporting pups born from stressed dams during pregnancy [75], also suggesting that the pathological programming effect of PRS on GABAergic neurotransmission is a key determinant of risk-taking behavior. Indeed, it has been shown that GABAergic drugs may be optimal to positively modulate risk-taking behavior [74]. A noteworthy observation is the reduction induced by PRS on the GABAA-α4 subunit in the ventral hippocampus, which was observed in both sexes. It has been shown that the GABA_A_-α4 subunit is primarily involved in alcohol motivational behavior [76]. Interestingly, previous studies on ethanol consumption in the PRS model have demonstrated that ethanol intake reduced HPA axis activity in PRS animals [77-79]. Therefore, these subunits emerge as promising targets for investigating the influence of PRS in the context of alcohol exposure.

### 4.3 PRS and Sex Effects on AMPA and GABA_A_ Receptors Ratio in Different Brain Regions

A significant novelty provided by our study is the assessment of the ratio between excitatory and inhibitory synaptic and extra-synaptic activity, mediated respectively by AMPAR and GABA_A_R subunits using protein density data in the context of PRS. Significant findings emerged predominantly in the dorsal hippocampus, with less pronounced effects in the ventral hippocampus and no significant changes in the prefrontal cortex. In the dorsal hippocampus, we observed a pronounced sex-dependent modulation of the AMPAR:GABA_A_R ratio at both synaptic and extra-synaptic levels. Specifically, PRS-exposed females exhibited increased synaptic and extra-synaptic inhibition *via* GABA_A_R subunits, suggesting a shift in the ionotropic GABAergic neurotransmission balance compared to males and control females. This shift may contribute to the sex-specific vulnerability observed in PRS rats, as extra-synaptic GABA_A_R subunits are targeted by neurosteroids that regulate this inhibitory pathway [80]. Consequently, in PRS-exposed females, there is a predominance of GABA_A_R-mediated inhibition at the synaptic level especially when the subunit GluA2 was excluded. This pattern corresponds with the elevated GluA2:GluA1 ratio observed in PRS females, which, combined with heightened synaptic GABA_A_R inhibition, may enhance the signal-to-noise ratio during learning, potentially improving cognitive performance [81]. In contrast, in the ventral hippocampus, the effects of PRS on the AMPAR:GABA_A_R balance appear to be similar across sexes. Therefore, the dorsal hippocampus seems to be a crucial region, where alterations in the excitation/inhibition balance could represent a mechanism of sex-dependent resilience in the context of early-life stress.

### 4.4 Principal Component Analysis (PCA) of PRS Effects on AMPA and GABA_A_ Receptor Subunits: Insights Cognition and Behavioral Response to Novelty

Overall, the characterization of the AMPA and GABA_A_ receptor subunits revealed a PRS-induced distinct dichotomy between the cognitive processes, supported by GluA2:GluA1 ratio and GABA_A_-α1 subunits modifications in the dorsal hippocampus and prefrontal cortex, and the behavioral response to novelty and adverse environment, bolstered by γ2 and GluA1 subunits changes in the ventral hippocampus. Consistently, Principal Component Analysis (PCA) conducted across the three distinct brain regions studied effectively summarized the global biochemical profile induced by PRS that is in association with the behavioral outcomes in both sexes. Specifically, PCA analysis showed that in the ventral hippocampus, associated with stress response regulation, the main neurochemical distinction was observed between PRS-exposed and control males and our results highlighted that PRS-exposed males show reduced risk-taking behavior. In contrast, in the dorsal hippocampus, which is related to cognitive performance, the main neurochemical comparison was identified between PRS-exposed females and males with PRS-exposed females showing improved cognitive performance associated with an increased risk-taking behavior in an adverse environment. Conversely, PRS-exposed males showed impaired cognition associated with reduced risk-taking behavior. This is consistent with existing literature indicating that stress-induced reductions in emotion-related behavior in animal models and in humans may lead to decreased cognitive flexibility [82]. Therefore, it is essential to consider the intricate interplay between emotions and cognitive function when interpreting behavioral data. In this context, the neurochemical distinctions in the subunit’s distribution pattern observed in the PRS rat model could offer valuable insights into understanding the neurodevelopmental trajectories involving the excitatory and inhibitory balance in the behavioral programming induced by PRS.

## CONCLUSION

Our findings highlight the critical role of brain excitation and inhibition levels in shaping synaptic responses to early environmental stimuli, which play a key role in the development of cognition and behavioral reactivity to novelty during sensitive periods. Importantly, our data strongly suggest that ELS induces enduring behavioral changes by altering the balance between AMPA excitatory and GABA_A_ inhibitory synaptic activity in a sex-dependent manner. Specifically, and although pharmacological validation is needed, biochemical analyses reveal that the changes in the GABA_A_-α1 subunit and AMPA-GluA2:GluA1 ratio in the dorsal hippocampus and prefrontal cortex may be associated with cognitive performance, while alterations in γ2 and GluA1 subunits observed in the ventral hippocampus might be related to response to novel and adverse environments. Consequently, our findings indicate that increased synaptic inhibition and reduced excitatory noise may underlie the enhanced recognition memory associated with an increased risk-taking behavior observed both in PRS females and in control males. Finally, we showed that ELS defeminizes and demasculinizes, inducing a sex-specific profile dependent on brain structure and behaviors, underscoring the importance of considering the interaction between early-life events and gender as a crucial element in applying precision medicine to psychiatric disorders.

## Figures and Tables

**Fig. (1):**
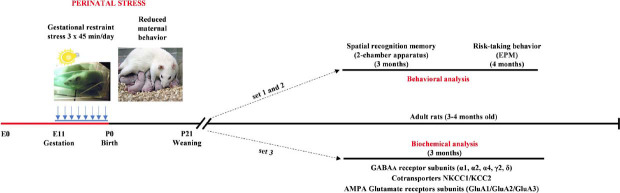
Experimental design. Male and female CONT and PRS rats, aged 3-4 months, were used for behavioral and biochemical analysis. Separate sets of animals were used for the two behavioral tests as well as for the biochemical analysis. Specifically, animals designated for biochemical analysis were behaviorally naïve. Biochemical analysis was performed in the hippocampus (ventral and dorsal) and the prefrontal cortex.

**Fig. (7):**
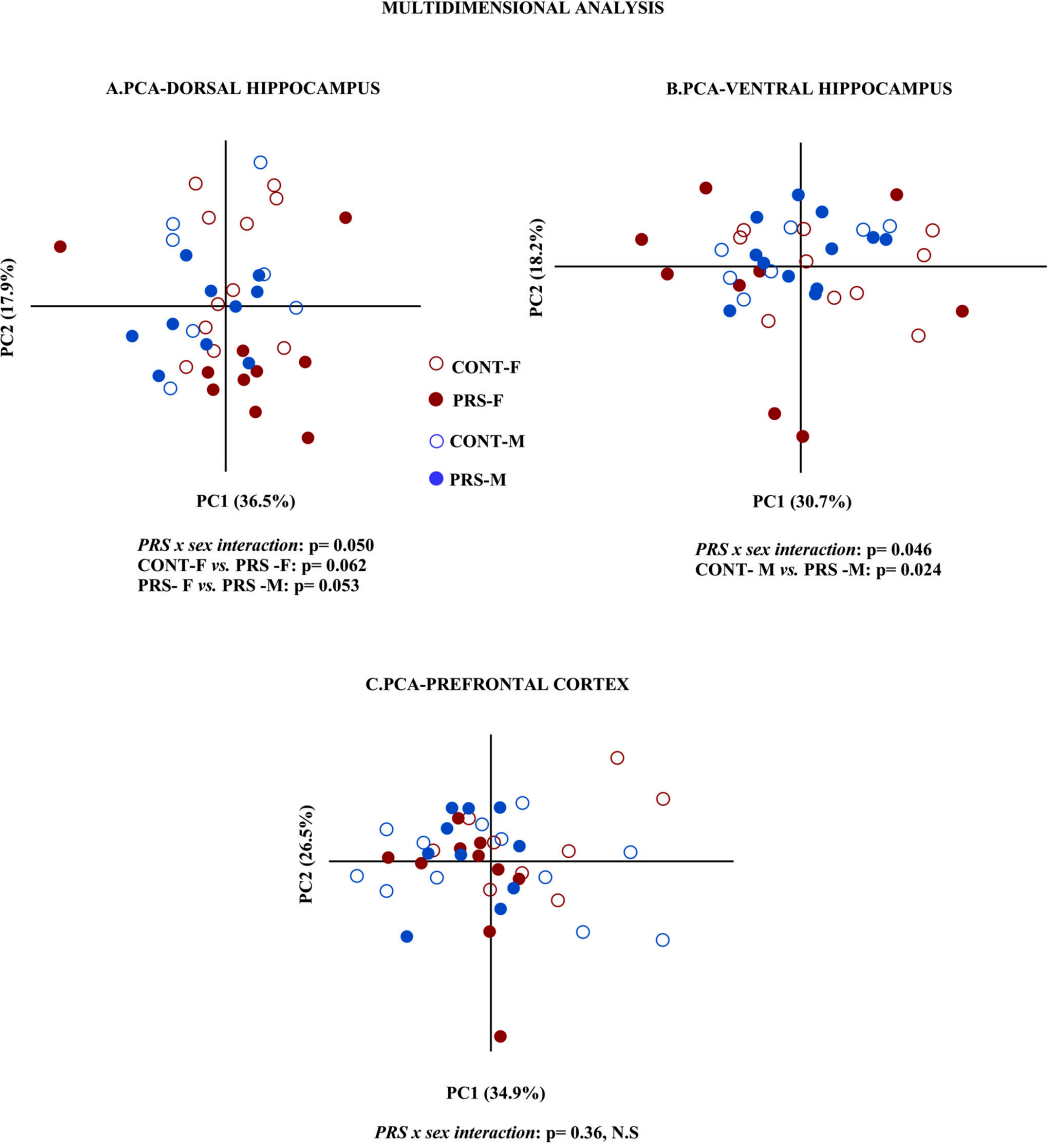
Multidimensional analysis. PCA-Principal component analysis in PRS and CONT unstressed rats of both sexes of the protein data set (AMPAR GluA1, GluA2, GluA3 subunits and the resulting GluA2:GluA1 ratio; GABA_A_ receptor α1, α2, α4, γ2 and δ subunits; NKCC1, KCC2 and their resulting NKCC1: KCC2 ratio). (**A**) Dorsal hippocampus, (**B**) ventral hippocampus, and (**C**) prefrontal cortex. PERMANOVA analysis was used to test group differences and the effects of variables in a multivariate manner.

## Data Availability

The data and supportive information are available within the article.

## LIST OF ABBREVIATIONS

PRS: Perinatal Stress
ELS: Early-life Stress
HPA: Hypothalamic-pituitary-adrenal
LTP: Long-term Potentiation
LTD: Long-term Depression
EPM: Elevated Plus Maze
